# Alginate-coated cobalt ferrite nanocomposite for preconcentration of heavy metals using dispersive solid phase extraction

**DOI:** 10.1039/d5ra10098d

**Published:** 2026-03-03

**Authors:** Maheen Khan, Jameel Ahmed Baig, Farah Naz Talpur, Saima Perveen, Hassan Imran Afridi, Khalil Akhtar, Nadeem Raza

**Affiliations:** a National Centre of Excellence in Analytical Chemistry, University of Sindh Jamshoro 76080 Pakistan; b Department of Chemistry, College of Science, Imam Mohammad Ibn Saud Islamic University (IMSIU) P.O. Box 90950 Riyadh 11623 Saudi Arabia JAMughal@imamu.edu.sa

## Abstract

Environmental pollution is becoming increasingly severe due to the widespread release of harmful substances, including heavy metals (HMs). HMs are of particular concern because they persist in the environment and bioaccumulate in living organisms, resulting in various health complications and ecological damage. In the current work, cobalt ferrite nanoparticles (CoFe_2_O_4_-NPs) were synthesized *via* a green sol–gel method using an aqueous extract of *M. indica* leaves. The synthesized CoFe_2_O_4_-NPs were used to fabricate an alginate-coated nanocomposite (Alg/CoFe_2_O_4_-NC) *via* an *in situ* method. The synthesized CoFe_2_O_4_-NPs and Alg/CoFe_2_O_4_-NC were characterized using advanced analytical techniques to assess their crystalline structure, crystallite size, degree of crystallinity, surface functionality, morphology, topography, surface charge, stability, and zeta size distribution. Alg/CoFe_2_O_4_-NC was employed to develop a novel dispersive solid-phase extraction (DSPE) method for simultaneous detection of cadmium (Cd), manganese (Mn), nickel (Ni), and lead (Pb) by flame atomic absorption spectrometry. Many experimental factors, including pH, nanocomposite dose, sample volume, eluent composition, eluent concentration, and eluent volume, were examined to maximize efficiency. The developed Alg/CoFe_2_O_4_-NC/DSPE/FAAS method showed limits of detection (LODs) for Cd (0.047 µg L^−1^), Mn (0.23 µg L^−1^), Ni (0.028 µg L^−1^), and Pb (0.107 µg L^−1^).

## Abstract 2

A natural polymeric ferrite adsorbent enables efficient dispersive solid-phase extraction, providing a rapid, reusable, and environmentally friendly analytical approach for the sensitive preconcentration of trace heavy metals from aqueous samples.

## Graphical abstract

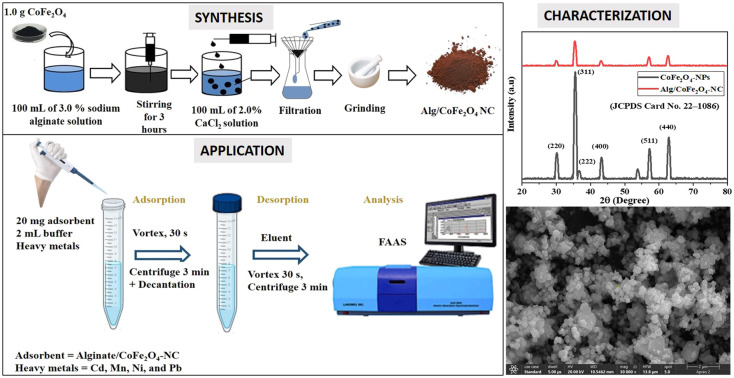

## 1. Introduction

Among the major environmental challenges worldwide, improving water quality is highly important, as water resources are becoming increasingly scarce due to climate change, population growth, and rising demand for water in agriculture and industry.^1^ However, the dumping of inorganic and organic toxins, mixed with disinformation and ignorance in their handling, can lead to inevitable damage to marine life. Environmental contaminants, such as heavy metals (HMs), dyes, and pharmaceutical residues, are increasing in quantity daily. Among these, the analysis of HMs is very crucial for environmental protection, food safety, and the purity of materials.^2,3^ In the field of agriculture, controlling environmental pollution and investigating HMs are also of great importance.^3,4^

Many instrumental techniques have been developed and employed for the quantitative measurement of HMs, and the most widely used is Flame atomic absorption spectrometry (FAAS) due to its affordability, trace-level detection, and ease of use. The preconcentration phase is important for quantifying trace levels of HMs in environmental samples, as it is challenging to determine them directly by FAAS due to their trace levels.^5^ HMs were determined using a variety of techniques, including flotation,^6^ electrochemical deposition,^7^ liquid–liquid extraction (LLE),^8^ co-precipitation,^9^ cloud point extraction,^10^ dispersive solid phase extraction (DSPE),^11^ and ion exchange.^12^

To improve extraction efficiency, a variety of nanomaterials have been used as sorbents for DSPE. Ferrite nanoparticles (FNPs), on the other hand, have demonstrated the greatest potential for effectively eliminating HMs from dietary and environmental samples. This is because of their advantageous characteristics, such as their high chemical reactivity and large surface area, which enable them to effectively extract HMs from the sample matrix.^13^

Developing a synthesis method capable of producing nanosized materials with superior magnetic performance compared to bulk materials is necessary to meet these demands. Spinel ferrite nanoparticles may be synthesised using a variety of wet chemical techniques, such as chemical co-precipitation, sol–gel, hydrothermal, solvothermal, thermal decomposition, and microwave combustion. These methods produce nanometer-sized particles at comparatively low temperatures and are cost-effective, straightforward, and time-efficient. They are therefore often employed in the production of magnetic spinel ferrite nanoparticles.^13^

The sol–gel auto-combustion approach is regarded as one of the most practical and efficient of these techniques as it uses a moderate reaction temperature (80–100 °C) and a quick powder synthesis procedure. It is a one-step process that produces a fine, fluffy, and voluminous powder. The procedure involves creating a sol, which is then gelled and burned in a heated solution containing metal salts and an appropriate fuel. The type of fuel, fuel-to-metal nitrate ratio, pH, and annealing temperature are among the preparatory conditions and factors that significantly impact the process efficiency and the quality of the final nanosized powder.^14,15^

Additionally, functionalized magnetic nanoparticles, such as cobalt ferrite nanoparticles (CoFe_2_O_4_-NPs) incorporated into biopolymers like alginate, have significant potential as support materials for HM detection. This composite integrates the excellent magnetic and mechanical properties of CoFe_2_O_4_-NPs with the biocompatibility and film-forming ability of alginate. As a result, such materials have attracted considerable attention for applications in wastewater treatment, biocatalysis, and bioremediation. Nevertheless, limited recovery of HMs results from the poor adsorption capacity of FNPs.^16^ Therefore, certain chemical modifications using natural or synthetic polymers to create nanocomposites can enhance their properties.^17^ Because of their structural porosity, ion-exchange capacity, hydrophilicity, and the active chemical properties of carboxyl and hydroxyl groups for binding HMs, natural polymers, primarily polysaccharides such as alginate, are preferred.^18^

The current study aims to synthesize CoFe_2_O_4_-NPs as the building block for the fabrication of an alginate-coated cobalt ferrite nanocomposite (Alg/CoFe_2_O_4_-NC). The Alg/CoFe_2_O_4_-NC/DSPE/FAAS method was used to assess HMs, such as Cd, Mn, Ni, and Pb. Advanced analytical methods were used to fully characterize the CoFe_2_O_4_-NPs and Alg/CoFe_2_O_4_-NC to investigate their crystalline structure, crystalline size, degree of crystallinity, surface functionality, morphology, topography, surface charge, stability, and zeta size distribution. The developed Alg/CoFe_2_O_4_-NC/DSPE/FAAS method was employed to simultaneously detect trace amounts of Cd, Mn, Ni, and Pb in tap water. Excellent results were obtained by optimizing the experimental parameters. The Alg/CoFe_2_O_4_-NC/DSPE/FAAS method was used to evaluate HM residues in tap water. Compared to the technique that detects each element independently, the method saves time and resources by detecting numerous HMs in a single run.

## 2. Experimental procedures

### 2.1 Chemicals

Analytical-grade standards and chemicals were employed in all the experimental studies. Cobalt chloride (CoCl_2_) ammonia (NH_3_), ethanol (C_2_H_6_O), Alginate(C_6_H_10_O_7_)calcium chloride (CaCl_2_) Sodium Nitrate(NaNO_3_) Sodium dihydrogen phosphate (NaH_2_PO_4_) and phosphoric acid (H_3_PO_4_) were used for pH 2, acetic acid (CH_3_COOH) and ammonium acetate (CH_2_COONH_4_) for pH 4–6, disodium hydrogen phosphate (Na_2_HPO_4_) and sodium dihydrogen phosphate for pH 6.5–7.5, and ammonia (NH_3_) and ammonium chloride (NH_4_Cl) for pH 8–10 and the standard stock solutions of Cd, Mn, Ni, and Pb of 1000 mg L^−1^ were also purchased from Sigma-Aldrich, Louis, USA. The deionized water was obtained from the ultra-purification system available at the National Centre of Excellence in Analytical Chemistry, University of Sindh, Jamshoro.

### 2.2 Green synthesis of cobalt ferrite nanoparticles

For the green synthesis of CoFe_2_O_4_-NPs, a 500 mL conical flask was taken and filled with a 100 mL solution mixture of Fe(NO_3_)_3_·9H_2_O and CoCl_2_·6H_2_O in 2 : 1 M respectively. The mixture was heated at 80 °C. Then, with the help of a burette, 50 mL of the produced *M. Indica* leaves extract was added dropwise to the resulting solution. The solution gradually formed a suspension, indicating successful nanoparticle synthesis. After 2 hours, the suspension was allowed to cool and filtered through a Whatman No. 1 filter paper. The resultant material was washed repeatedly with ethanol and deionized water, then dried in air. CoFe_2_O_4_-NPs were obtained by the resultant material, which was calcined for two hours at 500 °C and ground into a fine powder using a pestle and mortar.^19,20^

### 2.3 Fabrication of alginate-coated cobalt ferrite nanocomposite (Alg/CoFe_2_O_4_-NC)

The fabrication of Alg/CoFe_2_O_4_-NC was carried out by applying the reported procedure.^18^ First, 100 mL of a 3% (w/v) sodium alginate solution was prepared and mixed with 1 g of CoFe_2_O_4_-NPs. The mixture was stirred for three hours. After that, 100 mL of a 2% (w/v) CaCl_2_ solution was added dropwise to the mixture, which was then left overnight. The resultant material was filtered, thoroughly washed with distilled water to remove calcium impurity, dried for 24 hours at 30 °C, and then crushed into a fine powder of Alg/CoFe_2_O_4_-NC.

### 2.4 Instrumentation

The pH of the solutions was maintained *via* an Ion 6 meter (Eutech, Malaysia). XRD (Bruker D8-Advanced XRD equipment, Bruker, AXS INC., Wisconsin-USA) was employed to study crystalline structure, size, and degree of crystallinity of CoFe_2_O_4_-NPs and Alg/CoFe_2_O_4_-NC. FESEM and EDX (JEOL, JSM-7600F, Japan) were used to study the surface morphology and elemental composition of Alg/CoFe_2_O_4_-NC, respectively. The Atomic force microscopy (AFM) Nano-Scope V, Bruker, Germany) Instruments were used to study the surface topography of Alg/CoFe_2_O_4_-NC. A Fourier transform infrared (FTIR) spectrometer from Thermo Electron Scientific (Madison, WI, USA) was used to identify functional groups of the synthesized Alg/CoFe_2_O_4_-NC. The electro-kinetic potential and zeta size of Alg/CoFe_2_O_4_-NC were analyzed using a zeta potential analyzer (ELSZ-2000). HMs were analyzed using a flame atomic absorption spectrophotometer (Hitachi 180–50) bought from Hitachi High Technologies Corporation, Japan. Single-element hollow cathode lamps were employed for the determination of Cd, Mn, Ni, and Pb.

### 2.5 DSPE procedure

Alg-CoFe_2_O_4_-NC was used as the adsorbent in the DSPE technique to measure Cd, Mn, Ni, and Pb, according to the reported method. 200 mL of a solution that included Cd, Mn, Ni, and Pb was mixed with 100 mg of Alg-CoFe_2_O_4_-NC. Using suitable buffer solutions, the suspension pH was adjusted to 2.0–8.0. The suspensions were separated into several 50 mL centrifuge tubes after being vortex-mixed for 30 seconds. The samples were centrifuged at 3000 rpm for 3 min, after which the supernatant was discarded. Afterward, 2 mL of 1.0 M HNO_3_ was added as the eluent to the Alg/CoFe_2_O_4_-NC loaded with HMs. The mixture was vortexed for 30 seconds, then centrifuged at 3000 rpm for 3 minutes. Finally, the resulting supernatant was collected and analyzed by FAAS for HM determination.^21^

### 2.6 Sampling

The tap water samples were collected from the University of Sindh, Jamshoro. The samples were filtered and analyzed using the Alg/CoFe_2_O_4_-NC/DSPE/FAAS method.

## 3. Results and discussion

### 3.1 Characterization of Alg/CoFe_2_O_4_-NC

**Fig. 1:**
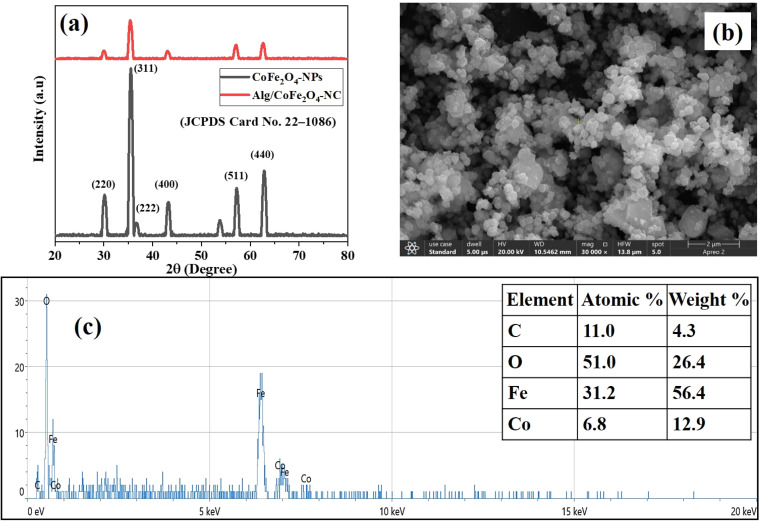
(a) XRD spectra of CoFe_2_O_4_-NPs and Alg/CoFe_2_O_4_-NC, (b) SEM image, and (c) EDX spectrum of synthesized CoFe_2_O_4_-NPs.

The XRD technique was used to study the structure, size, and crystallinity of the synthesized materials, and the results are shown in Fig. 1(a). In the XRD spectrum of CoFe_2_O_4_-NPs, all the peaks relate to the characteristic peaks of the cubic spinel lattice of CoFe_2_O_4_ (JCPDS File No. 22-1086), indicating that the samples have a single-phase spinel structure. Additional, diffraction peaks at 2*θ* values of 30.7, 35.9, 37.4, 43.6, 57.6, 63.1 resemble to the crystal planes (220), (311), (222), (400), (422), (511), (440), correspondingly and one additional peak was detected as an impurity may be due to formation of α-Fe_2_O_3_ at 34.2 which corresponds to 104 (JCPDS No. 33-0664).^22^ Furthermore, the XRD pattern of Alg/CoFe_2_O_4_-NC exhibited diffraction peaks similar to those of CoFe_2_O_4_ nanoparticles. It was also noted that no additional peaks corresponding to the biopolymer were found in the 2*θ* range of 20–80°, which confirms the successful synthesis of Alg/CoFe_2_O_4_-NC.^23,24^ The average crystallite sizes, calculated using the Debye–Scherrer equation, were 8.8 and 39.5 nm, respectively. Additionally, the sharp diffraction peaks point toward a high degree of crystallinity. The crystallinity (%) of CoFe_2_O_4_-NPs and Alg/CoFe_2_O_4_-NC was 92% and 83%, respectively. The decrease in the crystallinity (%) of Alg/CoFe_2_O_4_-NC confirmed its successful fabrication.^25^

The surface morphology of Alg/CoFe_2_O_4_-NC was examined using SEM, as shown in Fig. 1(b). The SEM study of Alg/CoFe_2_O_4_-NC revealed that the alginate matrix coating produced a very rough surface, altering particle morphology and increasing particle size. The surface area of Alg/CoFe_2_O_4_-NCs is further increased by their rougher surface.^26^ As a result, the produced nanocomposite may be shown to be successful in adsorbing HMs.

The EDX analysis further confirmed the material composition, as shown in Fig. 1(c). The atomic % indicated that dominant peaks for Fe (31.2%) and Co (6.8%) correspond to the cobalt ferrite framework, while the presence of C (11.0%) indicates the incorporation of the alginate matrix. The high oxygen content (51.0%) aligns with the organic material and ferrite components. These results collectively demonstrate the effectiveness of Alg/CoFe_2_O_4_-NC as a support material with its structural and compositional properties that promote stability and functionality.^27^

**Fig. 2:**
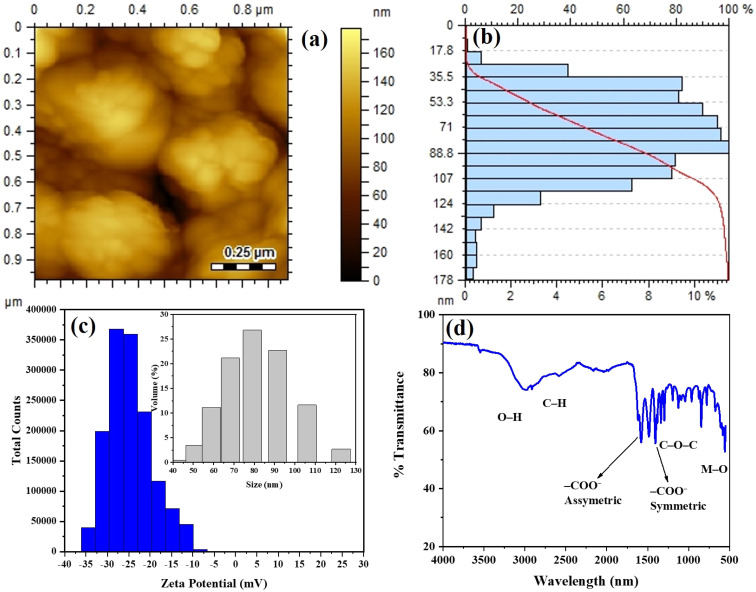
(a) AFM microscopy image, (b) size distribution plot, (c) zeta potential with zeta size, and (d) FTIR of synthesized Alg/CoFe_2_O_4_-NC.

The AFM was employed to study the surface topography of Alg/CoFe_2_O_4_-NC (Fig. 2(a)). The Alg/CoFe_2_O_4_-NC appeared agglomerated. The Alg/CoFe_2_O_4_-NC presents a smooth surface, suggesting a uniform polymer matrix without notable interruptions or inclusions.^28^ Further, the size distribution of the Alg/CoFe_2_O_4_-NC ranged from 17.8 to 178 nm, with an average particle size of 48.5 nm (Fig. 2(b)).

Surface charge and the attractive or repulsive interactions between CoFe_2_O_4_-NPs in aqueous solution were investigated using a zeta potential analyzer. A ZP value of > ± 30 mV generally indicates repulsive forces that stop particles from aggregating. The average ZP of the produced Alg/CoFe_2_O_4_-NC is −32.31 ± 4.9 mV, as shown in Fig. 2(c), confirming remarkable stability. Moreover, the average zeta size of Alg/CoFe_2_O_4_-NC was 60.3 ± 4.30 nm (see Fig. 2(c)).

FTIR studied the presence of functional groups, and the spectrum of Alg/CoFe_2_O_4_-NC is shown in Fig. 2(d). The results confirmed the successful incorporation of CoFe_2_O_4_-NP into the alginate matrix and the interaction between them. A broad absorption band observed in the region 3200–3500 cm^−1^ is attributed to the O–H stretching vibrations of hydroxyl groups in alginate and adsorbed moisture. Another absorption band around 2920–2850 cm^−1^ resembles the C–H stretching vibrations of aliphatic groups in the alginate backbone. Further, the strong absorption band near 1600–1630 cm^−1^ is due to the asymmetric stretching vibration of carboxylate groups (–COO^−^) present in alginate, while the band observed around 1400–1420 cm^−1^ relates to the symmetric stretching of –COO^−^ groups. The small shift and change in peak intensity of the obtained peaks, compared with those of pure alginate reported in our previous study,^29^ indicate coordination interactions between alginate carboxyl groups and metal ions on the CoFe_2_O_4_ surface. Moreover, the peaks appearing in the range 1000–1100 cm^−1^ are attributed to C–O–C and C–O stretching vibrations of the polysaccharide structure of alginate.^30^ It was noted that the characteristic absorption bands of metal–oxygen (Fe–O and Co–O) stretching vibrations observed below 600 cm^−1^, which confirmed the presence of Alg/CoFe_2_O_4_-NC.

### 3.2 Optimization of DSPE factors

#### 3.2.1 Effect of pH

**Fig. 3:**
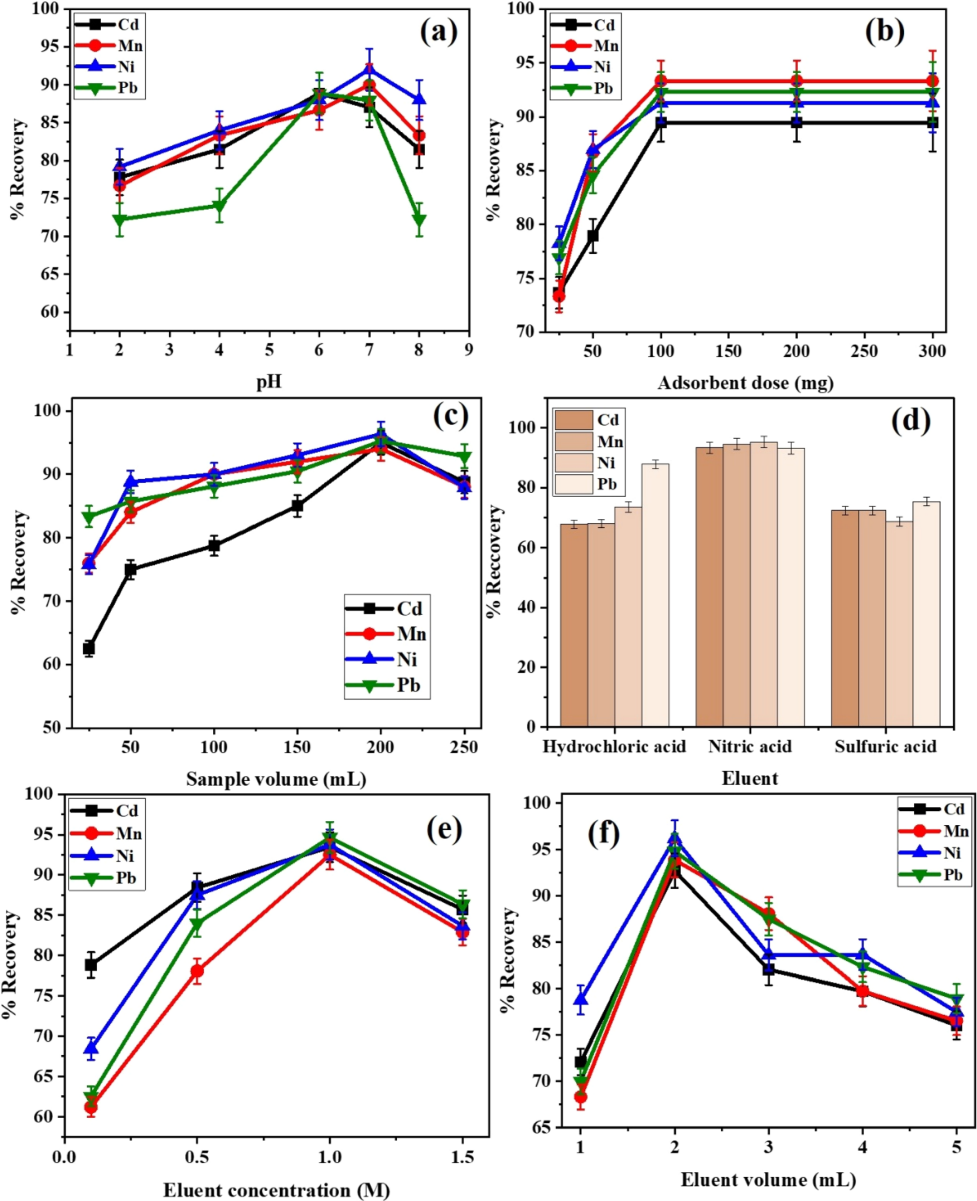
Optimization of (a) pH, (b) dosage, (c) sample volume, (d) eluent composition, (e) eluent concentration, and (f) eluent volume of % recoveries of HMs using Alg/CoFe_2_O_4_-NC based DSPE method.

The pH level plays a significant role in separation and preconcentration studies, as it governs the adsorption capacity of the adsorbent (Alg-CoFe_2_O_4_-NC) for HMs. The impact of pH on the adsorption of Cd, Mn, Ni, and Pb was investigated over the pH range of 2.0 to 8.0. The results in Fig. 3(a) showed that HM retention was higher at pH 7. Therefore, pH 7 is selected for the simultaneous analysis of Cd, Mn, Ni, and Pb.^24^

#### 3.2.2 Influence of adsorbent dose

The influence of Alg/CoFe_2_O_4_-NC dosage on the determination of HMs using the DSPE method was investigated at pH 7 by varying the dosage from 20 to 300 mg. The recoveries of HMs increased with an increase in the adsorbent dose up to 100 mg, as shown in Fig. 3(b). Beyond this amount, the recoveries remained nearly constant, indicating that sufficient active adsorption sites were available at 100 mg of Alg/CoFe_2_O_4_-NC for effective HMs adsorption. Based on these results, 100 mg of Alg/CoFe_2_O_4_-NC was selected as the optimal adsorbent dose for the determination of HMs using the developed DSPE method.

#### 3.2.3 Influence of sample volume

By varying the sample volume from 25 to 300 mL while maintaining the same experimental conditions, the impact of sample volume on HM recovery was examined. According to the findings shown in Fig. 3(c), HM recovery increased with increasing sample volume up to 200 mL, then declined as the sample volume increased further. The findings showed that at lower sample volumes, there may not be enough HMs to fully utilize the adsorbent's capacity, leading to poor recovery. The recoveries of studied HMs were improved by increasing the sample volume because more HMs are available to interact with the numerous carboxyl functional groups on the Alg/CoFe_2_O_4_-NC surface. However, the adsorbent became saturated, leaving insufficient active sites to bind all the HMs in the solution when the sample volume exceeded 200 mL. As a result, recovery decreased with larger sample sizes. Consequently, 200 mL was chosen as the optimal sample volume, as it provides the best balance between adsorbent capacity and analyte availability.

#### 3.2.4 Selection of best eluent

The findings of a study on several acids, including HNO_3_, HCl, and H_2_SO_4_, as possible eluents for the elution of HMs are shown in Fig. 3(d). Because of its potent oxidizing properties and capacity to effectively disrupt the linkage between HMs and the adsorbent, HNO_3_ was shown to produce the maximum recovery of HMs. Fig. 3(e) displays the findings of an examination into the concentration of the chosen eluent, which ranged from 0.1 to 1.5 M. Up to 1.0 M, it was observed that the recoveries of HMs rose as the eluent concentration increased. No noticeable increase was observed above this concentration, which may be due to matrix effects or saturation of the desorption efficiency. As a result, the ideal eluent concentration of 1.0 M was chosen. Additionally, Fig. 3(f) displays the results of optimizing the eluent volume over the range of 1–5 mL. Due to adequate desorption of the residual HMs with minimal dilution, 2.0 mL yielded the highest recoveries. Thus, the ideal eluent was determined to be 1 mL of 1.0 M HNO_3_. The preconcentration factor (PF; as the ratio of sample volume to eluent volume) was determined to be 100 based on an optimum sample volume of 200 mL and an eluent volume of 2.0 mL.

#### 3.2.5 Interference and reusability

The influence of interfering species may considerably affect the recoveries of analytes (Cd, Mn, Ni, and Pb) by interacting with them or the surface of the Alg/CoFe_2_O_4_-NC.^31^ The effectiveness of the Alg/CoFe_2_O_4_-NC/DSPE/FAAS method was evaluated by determining the recoveries of HMs (200 µg L^−1^) in the presence of interfering species such as Al^3+^, CH_3_COO^−^, Cl^−^, CO_3_^−^, Co^2+^, Mg^+2^, SO_4_^2−^, Na^+^, PO_4_^3−^, and K^+^, at concentrations 100 times higher than those of the target HMs. Each interfering species was studied individually by adding it separately to the sample solution in the presence of the target analytes. The results presented in Table 1 show that these foreign species did not significantly affect the recoveries of Cd, Mn, Ni, and Pb. These findings confirm the accuracy, selectivity, and efficiency of the developed Alg-CoFe_2_O_4_-NC/DSPE/FAAS method for the determination of HMs in real samples.

**Table 1:** Influence of foreign species on the determination of HMs by Alg/CoFe_2_O_4_-NC-based SPE-FAAS method

Foreign species	Added salts	% Recovery			
		Cd	Mn	Ni	Pb
Al^3+^	Al_2_(SO_4_)_3_	92.5	95.1	93.7	93.5
CH_3_COO^−^	CH_3_COONa	91.4	93.4	94.2	94.2
Cl^−^	NaCl	94.4	92.3	92.6	93.7
CO_3_^−^	Na_2_CO_3_	95.7	95.6	93.6	92.6
Co^2+^	Co(NO_3_)_2_	93.2	95.4	96.2	94.7
Mg^+2^	MgSO_4_	93.3	93.3	93.5	96.5
SO_4_^2−^	MgSO_4_	94.8	96.3	94.8	94.9
Na^+^	NaCl	91.2	96.4	95.9	95.5
PO_4_^3−^	Na_3_PO_4_	91.8	93.2	94.8	93.7
K^+^	KNO_3_	91.6	92.8	94.1	92.6

The reusability of the synthesized Alg/CoFe_2_O_4_-NC was assessed over five consecutive cycles using the same experimental procedure. The Alg/CoFe_2_O_4_-NC maintained its adsorption efficiency, with no significant decrease in HM recoveries, indicating excellent chemical and mechanical stability. These results demonstrate that Alg/CoFe_2_O_4_-NC can be reused effectively for at least five cycles with negligible loss in performance. Further, the functional groups responsible for HM binding remained intact, as evidenced by consistent recoveries and minimal leaching during desorption. These findings confirm that Alg/CoFe_2_O_4_-NC is a chemically robust and mechanically durable material, which makes it a reliable adsorbent for repeated DSPE applications.

#### 3.2.6 Analytical figures of merit, validation, and application

The analytical figures of merit were methodically assessed by employing the newly developed Alg/CoFe_2_O_4_-NC/DSPE method. Many parameters were examined under optimal circumstances, including linear concentration range, slope, enrichment factor (EF), correlation coefficient (*R*^2^), intercept, limit of detection (LOD, S/N = 3), and limit of quantification (LOQ, S/N = 10) for evaluating the effectiveness of the Alg/CoFe_2_O_4_-NC/DSPE method.^32^ The 3*σ*/*s* and 10*σ*/*s* formulae were used to determine the LODs and LOQs, respectively, where *s* is the slope of the calibration curve, and *σ* is the standard deviation of the blank measurements (*n* = 10).^33^ The LODs (µg L^−1^) obtained for the Alg/CoFe_2_O_4_-NC-based DSPE–FAAS method for Cd (0.047), Mn (0.23), Ni (0.028), and Pb (0.107). The enrichment factor was calculated as the ratio of the slope of the calibration curve obtained with DSPE to that obtained without DSPE.^34,35^ The EF values for Cd, Mn, Ni, and Pb were 98.4, 92.1, 92.0, and 92.3, respectively, which were approximately equal to the corresponding preconcentration factors (PF). These results demonstrate that the developed Alg/CoFe_2_O_4_-NC-based DSPE method is highly effective for the simultaneous determination of trace levels of HMs in real samples Table 2.

Analytical features of Alg/CoFe_2_O_4_-NC-based DSPE-FAAS method for the determination of HMs^a^

**Table:** 

Without DSPE				
Parameter	Cd	Mn	Ni	Pb
Dynamic range (µg L^−1^)	200–10 000	200–1000	200–20 000	400–20 000
Slope	0.0193	0.0102	0.0206	0.0262
Intercept	0.0276	0.0058	0.0313	0.0056
*R* ^2^	0.992	0.992	0.995	0.992
LOD (µg L^−1^)	1.39	1.02	0.79	1.02
LOQ (µg L^−1^)	4.6	3.42	2.6	3.41

a LOD (limit of detection); *R*^2^ (coefficient of determination; LOQ (limit of quantification).

**Table:** 

DSPE by Alg/CoFe_2_O_4_-NC				
Parameters	Cd	Mn	Ni	Pb
Dynamic range (µg L^−1^)	2–80	2–80	2–80	4–80
Slope	1.8994	0.9392	1.9853	2.4187
Intercept	0.012	0.036	0.0168	0.0043
*R* ^2^	0.998	0.998	0.997	0.998
LOD (µg L^−1^)	0.014	0.159	0.008	0.032
LOQ (µg L^−1^)	0.047	0.23	0.028	0.107

The real tap water samples were analyzed, and the results are shown in Table 3 for the detection of Cd, Mn, Ni, and Pb. Different concentrations were added, and the recovery and RSD values were calculated to assess accuracy and precision. Cd showed excellent recovery of 98–99% with minimal RSD. Mn and Ni also showed high recoveries (95–100%) and good precision. Lead showed recoveries of 96–97% and low RSD values. The results confirm that the analytical method is accurate, precise, and reliable for analyzing tap water.

**Table 3:** Real tap water analysis using the developed Alg/CoFe_2_O_4_-NC-based DSPE method for the quantitative determination of HMs

Analyte	Added (µg L^−1^)	Found (µg L^−1^)	Recovery (%)	RSD (%)
Cd	0	0.14	—	—
	50	49.4	98.7–99.2	0.16
	100	98.8	98.7–99.1	0.18
	200	199.5	99.5–99.8	0.15
Mn	0	BDL	—	—
	50	49.2	95.7–97.2	0.77
	100	99.4	97.5–99.3	0.90
	200	195.8	97.9–100.3	1.20
Ni	0	BDL	—	—
	50	48.7	97.4–98.5	0.54
	100	99.6	98.1–100.2	1.02
	200	198.7	97.7–100.4	1.35
Pb	0	BDL	—	—
	50	49.4	96.6–97.8	0.62
	100	99.1	96.9–99.6	1.35
	200	199.5	97.7–101.1	1.65

#### 3.2.7 Comparative study

The developed Alg/CoFe_2_O_4_-NC/DSPE/FAAS method demonstrates greater reliability compared to previously reported methods for the detection and quantification of HMs.^30,36–40^ The Alg/CoFe_2_O_4_-NC was employed as an adsorbent that provides high extraction efficiency, low limits of detection, and a high preconcentration factor. The results are summarized in Table 4, which highlights the excellent performance of the Alg/CoFe_2_O_4_-NC/DSPE/FAAS method in terms of efficiency, sensitivity, and analytical reliability for HM analysis. However, in a recent study,^30^ a DSPE–FAAS method using an alginate-integrated sodium ferrite nanocomposite (AG/Na_2_Fe_4_O_7_-NC) was reported, achieving lower LODs than the present Alg/CoFe_2_O_4_-NC-based method. This difference is scientifically reasonable and primarily arises from intrinsic crystallographic and surface-chemical differences between the two ferrite materials. Na_2_Fe_4_O_7_ exhibits a hexagonal structure, providing a higher density of surface-exposed Fe–O functional groups and stronger ion-exchange capacity, which favor enhanced adsorption of HMs with lower LODs. In contrast, CoFe_2_O_4_ holds a spinel structure in which cations are distributed within tetrahedral and octahedral positions, which results in relatively fewer accessible adsorption sites at the surface.

**Table 4:** Comparison of the developed Alg/CoFe_2_O_4_-NC-based DSPE method with reported studies for the quantitative determination of HMs^a^

Method	Sorbent	Detection technique	PF	LOD (µg L^−1^)	Ref.
mSPE	Luffa@TiO_2_	FAAS	50	0.13	36
SPM	Bi_2_WO_6_	FAAS	50	6.0	37
DSP-Me	ND@Bi_2_MoO_6_	FAAS	24.5	1.75	38
Mspe	MWCNTs@MgAl_2_O_4_@TiO_2_	FAAS	100	0.42	39
SPME	Nanodiamonds@NiCoFe-LDH	FAAS	25	0.621	40
DSPE	AG/Na_2_Fe_4_O_7_-NC	FAAS	250	0.0013–0.0063	30
DSPE	Alg/CoFe_2_O_4_	FAAS	100	0.047–0.23	Current study

a FAAS; flame atomic absorption spectroscopy, mSPE; magnetic solid phase extraction, SPME; solid phase microextraction, DSPE; dispersive solid phase extraction, Fe_3_O_4_@MBA nano-hybrid; magnetite Fe_3_O_4_@biosilica/alginate, MSPE; magnetic solid phase extraction, Fe-alg-MgO; iron alginate magnetic graphene oxide, Alg-Fe_3_O_4_; sodium alginate-coated magnetite.

Notably, both (AG/Na_2_Fe_4_O_7_-NC) and Alg/CoFe_2_O_4_-NC were synthesized using the same sol–gel method, which confirms that the observed variations originate from the intrinsic material properties rather than synthesis or experimental conditions. Despite slightly higher detection limits, the Alg/CoFe_2_O_4_-NC exhibits excellent extraction efficiency, magnetic separability, reproducibility, and operational stability, making it highly suitable for routine multi-element analysis in complex real samples. Therefore, the present method offers a balanced combination of sensitivity, robustness, and practical applicability.

## 4. Conclusion

CoFe_2_O_4_-NPs were successfully synthesized using *Mangifera indica* leaf extract, and then fabricated with sodium alginate powder to obtain Alg/CoFe_2_O_4_ nanocomposite. The synthesized nanocomposite was characterized, and FTIR confirmed the successful synthesis of CoFe_2_O_4_-NPs, with characteristic peaks of alginate and metal–oxygen stretching vibrations. Further, SEM and AFM analysis revealed a spherical shape. After combining with alginate, the surface becomes rough with irregularities, which is desirable for solid phase extraction, while XRD confirms the spinel phase of synthesized Alg/CoFe_2_O_4_-NC with crystallite size 39.5 nm, respectively. Alg/CoFe_2_O_4_-NC was employed to develop a novel DSPE method for the simultaneous detection of HMs by FAAS with excellent LODs (µg L^−1^) for Cd (0.047), Mn (0.23), Ni (0.028), and Pb (0.107). This developed method was successfully applied to measure HMs in tap water. Hence, Alg/CoFe_2_O_4_-NC offers efficient DSPE method and makes it a desirable means for analyzing HMs in tap water samples.

## Author contributions

The current study owes its conceptualization, supervision, project administration, and review/editing to the esteemed efforts of Prof. Dr Jameel Ahmed Baig, Prof. Dr Farah Naz Talpur, Dr Hassan Imran Afridi, and Dr Nadeem Raza. Ms Maheen Khan, a dedicated M.Phil scholar, played a pivotal role in data curation, formal analysis, methodology, and creating the original draft. The technical aspects, including software usage, investigation, and visualization, were skillfully handled by Saima Perveen and Khalil Akhtar.

## Conflicts of interest

All authors stated that they had no known financial or personal conflicts of interest that would have influenced the research. The current experimental investigations do not involve any human or animal contributors, either directly or indirectly.

## Data Availability

Data will be made available on request.
